# Improved grip myotonia in a patient with myotonic dystrophy type 1 following electroacupuncture therapy: A CARE-compliant case report

**DOI:** 10.1097/MD.0000000000021845

**Published:** 2020-09-11

**Authors:** Sang-Hoon Yoon, Jang-Hyun Baek, Jungtae Leem

**Affiliations:** a Chung-Yeon Central Institute; b Chung-Yeon Korean Medicine Hospital, Seo-gu, Gwangju; c Department of Neurology, Kangbuk Samsung Hospital, Sungkyunkwan University School of Medicine; d Research and Development Institute, CY Pharma Co., Gangnam-gu, Seoul, Republic of Korea.

**Keywords:** acupuncture, case report, electroacupuncture, myotonic dystrophy, traditional East Asian medicine

## Abstract

**Rationale:**

Myotonic dystrophy type 1 (DM1) is an autosomal-dominant disorder associated with a short life expectancy and various symptoms, including grip myotonia. Even though grip myotonia decreases quality of life, activities of daily living (ADLs), and work performance, very few interventions provide symptomatic relief.

**Patient concerns:**

In this case report, we present a patient with DM1 and gradually worsening grip myotonia. A 35-year-old woman developed grip myotonia at age 27. She had no underlying diseases or family history of relevant conditions, including DM1. She was unresponsive to medication for several years.

**Diagnosis:**

Her symptoms gradually worsened, and she was finally diagnosed with DM1 via genetic, neurologic, and laboratory testing in a tertiary hospital at age 32. She tried several medication therapies; however, she stopped medication at age 34 due a perceived poor response and several adverse events.

**Intervention:**

At the age of 35, she underwent 29 sessions (10 minutes per session) of electroacupuncture therapy on TE9 acupuncture point with 120 Hz electrical stimulation over 3 months.

**Outcomes:**

After 3 months, relaxation time after maximal voluntary isometric contraction decreased from 59 to 2 seconds with treatment. Her Michigan Hand Outcomes Questionnaire score improved (total score, 66.6–75.9; ADL sub-score, 59.7–73.6; function sub-score, 70–90; satisfaction sub-score, 75–91.7). Her Measure Yourself Medical Outcome Profile 2 score also improved from 4.33 to 2. There were no serious adverse events.

**Lessons:**

Electroacupuncture is a potential treatment modality and produced an immediate antimyotonic effect, and cumulative long-term treatment effect, in a patient with DM1 and grip myotonia. Other notable treatment outcomes included improving relaxation time, hand function, ADLs, and overall satisfaction. Electroacupuncture is a potential treatment modality for patients with DM1 and grip myotonia. Further prospective clinical studies are warranted to confirm this hypothesis.

## Abstract 2

Supplemental Digital Content is available in the text

## 1 Introduction

Myotonic dystrophy type 1 (DM1) is an autosomal-dominant disorder characterized by grip myotonia, facial weakness, ptosis, and distal muscle weakness.^[1]^ The prevalence of DM1, the most common inherited muscular dystrophy in adults, is estimated at 1/8000,^[2]^ but patients with DM1 can be a challenge to understand due to their considerable symptom heterogeneity and molecular etiology.^[3]^ A patient with DM1 may develop various symptoms such as cataracts, arrhythmias, and endocrine disorders^[4]^; furthermore, life expectancy is reduced by 70% due to cardiac and pulmonary complications.^[5]^ However, there is no effective treatment, and only a few interventions that may provide symptomatic relief.^[6]^

Acupuncture is a traditional East Asian medicine treatment modality that is considered efficacious, safe, and cost-effective for treating several chronic pain.^[7,8]^ Acupuncture is also widely used to treat movement disorders like Parkinson disease.^[9]^ We report on a patient with DM1 with gradually worsening grip myotonia who, after being unresponsive to medication for 2 years, experienced marked symptom improvement following electroacupuncture (EA) treatment. This study is written according to the Case report guidelines (CARE)^[10]^ (Supplement 1).

## 2 Case presentation

**Figure 1:**
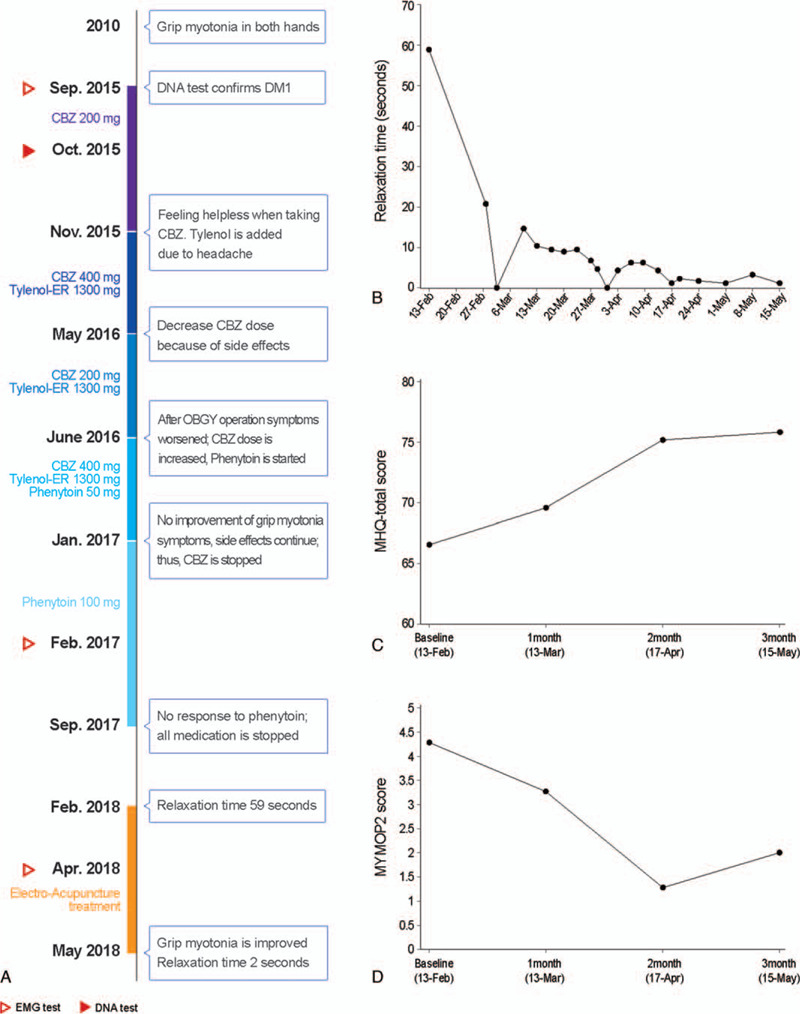
Timeline of the patient's progress. ADL = activities of daily living; CBZ = Carbamazepine; DM1 = Myotonic dystrophy type 1; MHQ = Michigan Hand Outcomes Questionnaire; MYMOP = Measure Yourself Medical Outcome Profile; s = seconds.

A 35-year-old woman developed grip myotonia at age 27. She had no underlying diseases or family history of relevant conditions, including DM1. Her symptoms gradually worsened, and she visited a tertiary hospital at age 32. A neurological examination revealed no focal neurological deficits, including weakness or muscle atrophy. Myotonia was observed only when gripping with both hands. Her laboratory tests were normal. Needle EMG showed increased insertional activity and a myotonic response with positive sharp waves and fibrillation potential. Genetic testing revealed *DM1* (CTG)_n_ with repeat lengths of 150. She was finally diagnosed with adult-onset DM1. She was started on carbamazepine (CBZ) 200 mg/d. Immediately after medication administration, her grip myotonia improved without discomfort. However, the symptoms returned after 6 hours. Despite CBZ, her hand grip relaxation time (RT) gradually increased over 1 year. The CBZ dose was increased to 400 mg/d; later, due to lethargy, it was changed to phenytoin 100 mg/d. Nevertheless, her symptoms were consequently aggravated, and she eventually stopped taking all medicine. Five months later, she visited our clinic with a chief complaint of bilateral grip myotonia (Fig. 1).

Her baseline RT after a maximal voluntary isometric contraction was 59 seconds. Figure 1 shows the time-course of the patient's condition. EA treatment was performed 29 times over 3 months. After insertion of acupuncture needles into the acupuncture point TE9 and 2 cm above TE9 at the extensor digitorum communis in the forearm, 10-Hz electrical stimulation was applied, with visible muscle twitching, for 10 minutes. The EA procedure is described in the STandards for Reporting Interventions in Clinical Trials of Acupuncture (STRICTA) checklist^[11]^ (Supplement 2).

Initially, she could repeatedly grasp and open her hands without grip myotonia immediately after EA treatment, although her symptoms generally returned within an hour. However, with additional acupuncture sessions, her symptom relief was prolonged. After the 24th treatment session, she maintained a normal state with good symptom control for up to 7 days. At this point, her treatment frequency was decreased to once a week.

Improved RT and symptoms were well maintained (Fig. 1). Her grip RT decreased from 59 seconds (1st session) before treatment to within 2 seconds after 29 treatment sessions over 3 months. A video clip of her hand motion is provided as Video-1. During EA treatment, she did not use any myotonia-related medication or engage in any other rehabilitation.

The Korean version of the Michigan Hand Outcomes Questionnaire (K-MHQ) is a hand-specific outcome instrument which assesses overall function, activities of daily living (ADLs), pain, work performance, aesthetics, and patient satisfaction.^[12]^ The K-MHQ was administered at monthly intervals to measure functional hand improvements. Over 3 months, there was an improvement in the K-MHQ total score (from 66.6 to 75.9), function score (from 70 to 90), satisfaction score (from 75 to 91.7), and ADL score (from 59.7 to 73.6) (Table 1).

**Table 1:**
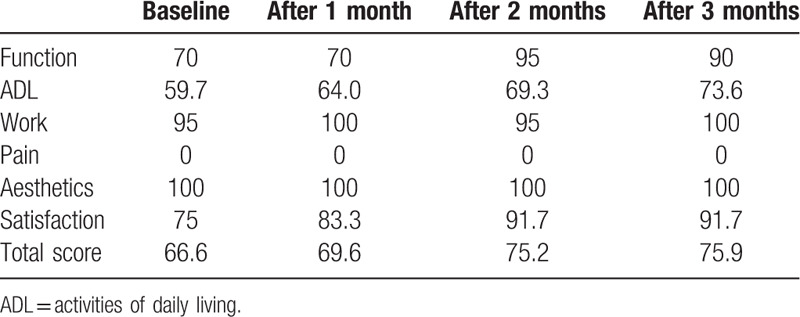
Change of Michigan Hand Outcomes Questionnaire score.

The Measure Yourself Medical Outcome Profile 2 (MYMOP2) is a patient-reported outcome measure where patients report any specific symptoms, activity limitations, and emotional states.^[13]^ The MYMOP2 can be utilized to assess physical, emotional, and social variables using a brief and simple questionnaire. Results are reported according to a seven-point Likert scale (from 0 to 6), and average scores are used. The most uncomfortable symptoms were described as “a symptom of not spreading well after holding her hand tight,” and her most uncomfortable activity was described as “uncomfortable when locking the buttons on clothes.” After treatment, her MYMOP2 scores for her 2 chief complaints decreased from 4.33 to 2 (Fig. 1).

There was no deterioration in EMG after EA treatment. Over the 3 months of treatment, no adverse effects were observed except for bruises, <2 cm in diameter, located at the EA treatment site. These bruises were monitored closely and improved within 1 week.

## 3 Patient's perspective

“I learned nail art two years ago. Nevertheless, it was difficult to pick up the bottle cap of the nail polish, so I could not do nail art. However, now it is possible to pick up the bottle cap, and I can do nail art again. Two days after the treatment, the stiffness does not come back, and the function is well maintained.”

## 4 Discussion

There is no prior report of improved grip myotonia symptoms in a patient with DM1 following EA treatment. In this case, the patient's grip myotonia had worsened despite a 2-year course of medication; however, it was markedly improved within 3 months following introduction of EA treatment. As there were no other interventions except for EA, the observed effects appear attributable to EA treatment. Also, her grip myotonia immediately disappeared after starting EA treatment; this observation also supports the efficacy of EA. Moreover, the long-term cumulative treatment effect also minimizes bias. However, the lack of long-term follow up is a limitation of this study. In DM1, myotonia is caused by skeletal muscle membrane hyper-excitability. Although the loss of the muscle-specific chloride channel or abnormal Na^+^ channel activity has been suggested to underlie hyperexcitability, the exact pathophysiological mechanism is unclear.^[14]^

Recently, the effects of mexiletine, a non-selective voltage-gated sodium channel blocker, has been reported to alleviate myotonia symptoms.^[15]^ However, if the myotonia symptoms do not respond to medication, no other treatments have been reported to date.

The therapeutic effect or mechanism of action of EA for myotonia in DM1 has not been reported. However, EA is widely used as a non-pharmacological intervention for spasticity,^[16]^ and EA is known to affect muscle hyper-excitability. In a previous post-stroke spasticity study, acupuncture increased the mean H-reflex recovery time within the antagonistic muscle of the spastic muscle.^[17]^ Mukherjee et al^[18]^ reported that, when EA was applied to the extensor muscle of the forearm, EMG signals of the flexor muscle (antagonistic muscle) were reduced. In an experimental study, EA reduced the hyper-excitability of motoneurons in the spinal cords of experimentally injured rats.^[19]^ Taken together, we hypothesize that acupuncture reduces the hyperexcitability of gamma and alpha motor neurons, and increases the inhibition of interneurons, thereby regulating the activity of spinal motor neurons.^[20]^ Additionally, EA treatment can help muscle regeneration by inhibiting myostatin gene expression.^[21]^ A fairly recent clinical study reported enhanced ankle dorsiflexor muscle strength after EA stimulation in normal anterior tibial muscles was reported.^[22]^ Thus, the mechanism may involve an increase in the stimulated muscle's strength after EA treatment, coupled with antagonistic muscle relaxation. However, the mechanism of spasticity and myotonia in DM1 is different, so additional experimental studies are needed.^[23]^

The treatment effects of EA can be interpreted as a warm-up effect through continuous activities. However, the warm-up effect does not persist, so the same level of myotonia is observed when measuring the next day.^[24]^ We believe that EA operates via a different mechanism from the warm-up effect because the treatment effect lasted for several days as the sessions accumulated.

Our patient only received EA treatment, and no other interventions were performed. Additionally, as grip myotonia improved immediately after EA treatment, symptom improvement appeared attributable to EA. To our best knowledge, this is the first report of the effects of EA in patient with DM1. A limitation of this study which deserves consideration is the lack of long-term follow up after the end of treatment. Future, well-powered, and prospective studies of the therapeutic effects and mechanisms of action of EA treatment are needed. However, this case suggests that EA could be a possible treatment for patients with DM1 and grip myotonia and may produce an immediate antimyotonia effect and long-term cumulative treatment effect characterized by improved relaxation time, improved hand function, improved ADL execution, and improved overall satisfaction.

## Acknowledgments

Thanks to the patient for allowing us to use medical information in this study.

## Author contributions

**Conceptualization:** Sang-Hoon Yoon.

**Methodology:** Sang-Hoon Yoon, Jungtae Leem.

**Supervision:** Jungtae Leem.

**Writing – original draft:** Sang-Hoon Yoon.

**Writing – review & editing:** Jang-Hyun Baek, Jungtae Leem.

## Supplementary Material

Supplemental Digital Content

## Supplementary Material

Supplemental Digital Content

## Supplementary Material

Supplemental Digital Content
