# Supplementary material for: Improved grip myotonia in a patient with myotonic dystrophy type 1 following electroacupuncture therapy: A CARE-compliant case report
Source: Medicine (Baltimore). 2020 Sep 11;99(37):e21845. doi: 10.1097/MD.0000000000021845 (PMC7489697; doi:10.1097/MD.0000000000021845)
Supplement: Supplemental Digital Content [file medi-99-e21845-s001.docx]

**Checklist for items in STRICTA 2010**

| **Item** | **Detail** |
| --- | --- |
| **1. Acupuncture rationale** | 1a) Style of acupuncture: The style of acupuncture used in this study was based on a recent study, as shown in 1b. |
|  | 1b) Reasoning for treatment provided, based on historical context, literature sources, and/or consensus methods, with references where appropriate: The authors performed acupuncture treatment with reference to Mukherjee et al.'s^1^ previous treatment of post-stroke spasticity and Takaoka et al.'s^2^ report that electro-acupuncture treatment regenerates muscle. |
|  | 1c) Extent to which treatment was varied: 1 case report; the treatment was the same in all sessions. |
| **2. Details of needling** | 2a) Number of needle insertions per subject per session (mean and range where relevant) : 4 |
|  | 2b) Names (or location if no standard name) of points used (uni/bilateral): TE9 (四瀆) and 2 cm above TE9, located at the extensor digitorum communis muscle in the forearm, bilateral |
|  | 2c) Depth of insertion, based on a specified unit of measurement, or on a particular tissue level : 1.5cm, muscle |
|  | 2d) Response sought (e.g. *de qi* or muscle twitch response): muscle twitch response |
|  | 2e) Needle stimulation (e.g. manual, electrical): Electrical (GP 304, Goodpl, Korea), 10 Hz. The intensity of the current was increased to a tolerable intensity. |
|  | 2f) Needle retention time: 10 minute. |
|  | 2g) Needle type (diameter, length, and manufacturer or material): Filiform needle; diameter: 0.25 mm, length: 30 mm; Dongbang Acupuncture Needle Company, Boryung-si, Korea. |
| **3. Treatment regimen** | 3a) Number of treatment sessions: 29 session over a period of 3 months. |
|  | 3b) Frequency and duration of treatment sessions: Average 2 sessions per week, 10 minutes per session |
| **4. Other components of treatment** | 4a) Details of other interventions administered to the acupuncture group (e.g. moxibustion, cupping, herbs, exercises, lifestyle advice) : no other intervention |
|  | 4b) Setting and context of treatment, including instructions to practitioners, and information and explanations to patients: This case was treated as an outpatient and every treatment was performed in an independent, single-person room. In addition to EA treatment, no other physical therapy, exercise therapy, or life teaching was given. Before each EA treatment, the therapist measured the relaxation time. When the patient felt pain during acupuncture, the practitioner reapplied EA an area where the pain was not felt. Electrical stimulation was applied until a sufficient muscle twitch response occurred. Patients were informed that bruising or pain may occur after treatment. |
| **5. Practitioner background** | 5) Description of participating acupuncturists (qualification or professional affiliation, years in acupuncture practice, other relevant experience): After graduating from a Korean Medicine college, the practitioner obtained a Korean Medicine Doctor’s license and has 8 years of experience in acupuncture practice. |
| **6. Control or comparator interventions** | 6a) Rationale for the control or comparator in the context of the research question, with sources that justify this choice : Not applicable because it is a case report. |
|  | 6b) Precise description of the control or comparator. If sham acupuncture or any other type of acupuncture-like control is used, provide details as for Items 1 to 3 above. : Not applicable because it is a case report. |

Note: This checklist, which should be read in conjunction with the explanations of the STRICTA items, is designed to replace [CONSORT 2010](http://www.consort-statement.org/consort-statement/3-12---methods/item5_interventions/)’s item 5 when reporting an acupuncture trial.

**References**

1. Mukherjee M, McPeak LK, Redford JB, Sun C, Liu W. The effect of electro-acupuncture on spasticity of the wrist joint in chronic stroke survivors. Arch Phys Med Rehabil. 2007;88:159–166.

2. Takaoka Y, Ohta M, Ito A, et al. Electroacupuncture suppresses myostatin gene expression: cell proliferative reaction in mouse skeletal muscle. Physiol Genomics. 2007;30:102–110.
